# Over-the-Loop Technique for Combined Anterior Cruciate Ligament Reconstruction and Modified Lemaire Tenodesis: Enhancing Rotational Stability With a Single Femoral Tunnel

**DOI:** 10.1016/j.eats.2025.103570

**Published:** 2025-05-07

**Authors:** Cristiano José Silva Manze, Roberto da Cunha Luciano, Camilo Partezani Helito, Sergio Marinho de Gusmão Canuto, Pedro Baches Jorge, Vitor Barion Castro de Padua, Diego Ariel de Lima

**Affiliations:** a BS Knee (Brazilian Storm) Research Group, São Paulo, Brazil; b Hospital Navegantes, Porto Seguro, Brazil; c Hospital de Clínicas da Universidade Federal de Uberlândia (HC-UFU), Uberlândia, Brazil; d USP (Universidade de São Paulo), Grupo de Joelho, Instituto de Ortopedia e Traumatologia, Hospital das Clínicas (HCFMUSP), Faculdade de Medicina da Universidade de São Paulo, São Paulo, Brazil; e Ortoclinica, Hospital de Ortopedia, Maceió, Brazil; f Santa Casa de Misericordia de São Paulo, São Paulo, Brazil; g Universidade de Marília (UNIMAR), Marília, Brazil; h Universidade Federal Rural do Semi-Árido (UFERSA), Mossoró, Brazil

## Abstract

This article describes a combined technique for anterior cruciate ligament reconstruction and modified anterolateral Lemaire tenodesis using a single femoral tunnel. The approach uses quadrupled hamstring grafts (semitendinosus and gracilis) and an “over-the-loop” passage of the iliotibial band through the graft loop. This technique aims to enhance rotational stability, minimize the risk of tunnel convergence, and reduce technical complexity and associated costs. Biomechanical benefits include improved rotational control, reduced graft rerupture rates, and optimized postoperative rehabilitation. The simplicity and reproducibility of this technique make it an attractive option for orthopaedic surgeons treating high-risk patients or rotational sport athletes.

Anterior cruciate ligament (ACL) injuries are common and often cause instability. Isolated ACL reconstruction may not sufficiently control rotational instability, particularly in young, high-demand athletes or patients with a grade 2 to 3 pivot shift, increasing the risk of graft rerupture.^1^ To address this, extra-articular techniques such as lateral extra-articular tenodesis (LET) and anterolateral ligament (ALL) reconstruction have gained popularity, enhancing rotational control and reducing graft failure. However, their proximity to ACL fixation may cause technical challenges, increased costs, and complications.^2^^,^^3^

Single–femoral tunnel techniques for ACL and ALL fixation simplify surgery, reducing tunnel convergence and hardware use.^4^, ^5^, ^6^, ^7^ This article presents a combined technique for ACL reconstruction and modified Lemaire tenodesis using a single femoral tunnel. The approach uses a quadrupled ACL graft with passage of the iliotibial tract (ITT) “over the loop” of hamstring grafts at the tunnel exit, optimizing rotational control while minimizing complexity and material use.

## Surgical Technique

The complete technique is shown in Video 1; pearls and pitfalls, in Table 1; and advantages and disadvantages, in Table 2.

**Table 1:** Pearls and Pitfalls

Pearls
Accurate identification of candidates for combined ACL reconstruction and LET is essential, especially among young athletes with rotational instability.
An imaging examination should be performed for surgical planning, especially in revision cases.
Attention to tendon positioning is required: An Ethibond thread should be passed through the loop of the folded graft to facilitate smooth and controlled passage of the graft through the tibial and femoral tunnels.
A second Ethibond thread with a unique loop configuration is used to transport the ITT during the modified Lemaire technique, ensuring precise positioning after the graft passes through the femoral tunnel.
The hamstring grafts should be secured transversely to prevent slippage.
Caution is required during lateral femoral access: Identification of the lateral femoral epicondyle and Gerdy tubercle is essential (when in doubt, fluoroscopy should be used).
Attention is required for preparation of the ITT: An ITT with a width of at least 1 cm and length of 8 cm is required; this ensures the over-the-loop configuration, characteristic of the modified Lemaire technique.
Care is required when applying traction to the graft at the tibial tunnel exit: The surgeon should ensure that the loop of the hamstring grafts is flush with the femoral cortex.
For graft fixation in the femoral tunnel, the surgeon should apply gentle traction to the ITT stump to avoid excessive tension, which could increase lateral compartment pressure postoperatively.
Graft protrusion must be avoided: The surgeon should ensure that no part of the ACL graft protrudes from the lateral femoral condyle because this could cause friction and subsequent pain.
Pitfalls
Incorrect positioning of the femoral or tibial tunnels may compromise graft stability.
Excessive tension during fixation of the ITT may increase lateral compartment pressure.
Failure to correctly perform the over-the-loop passage of the ITT through the graft loop can lead to uneven tension distribution.
Incorrect graft length or configuration can lead to difficulties during fixation or postoperative complications.
The surgeon should avoid leaving protruding graft material or hardware because it may cause irritation or postoperative pain.

ACL, anterior cruciate ligament; ITT, iliotibial tract; LET, lateral extra-articular tenodesis.

**Table 2:** Advantages and Disadvantages

Advantages
ACL and LET reconstruction are combined using a single femoral tunnel, simplifying the procedure.
The risk of tunnel convergence is reduced, and the use of fixation devices is minimized.
Rotational stability is enhanced, reducing graft rerupture rates in high-risk patients.
The technique provides a reproducible and cost-effective option in resource-limited settings.
Disadvantages
The technique requires precise technical execution and familiarity with the over-the-loop modified technique.
There is potential for increased surgical time because of additional steps for graft preparation and fixation.
The technique may be technically challenging for surgeons without adequate training in combined reconstruction.
Excessive tension during fixation can lead to complications, requiring careful intraoperative adjustment.

ACL, anterior cruciate ligament; LET, lateral extra-articular tenodesis.

### Surgical Indications

The main surgical indications described for combined ACL reconstruction and ALL reconstruction are ACL revision surgery, physical examination findings of a grade 2 or 3 pivot shift, participation in sports involving pivoting mechanisms and/or high-level activity, ligamentous laxity, and Segond fracture. Secondary indications may also include chronic ACL injury, age younger than 25 years, and radiologic signs of lateral femoral condylar depression.^6^

### Necessary Materials for Procedure

The materials required for the procedure are as follows: 2 interference screws; 1 cannulated reamer or drill; 90° femoral guide (e.g., Chambat guide); 55° tibial guide; 2 guide pins (2 mm); tendon stripper; FiberWire (Arthrex, Naples, FL), Vicryl (Ethicon, Somerville, NJ), and/or Ethibond Excel (Ethicon) suture; and basic materials for arthroscopy.

### Graft Harvest

An incision of approximately 2 to 3 cm is made over the insertion of the pes anserinus tendons, medially to the tibial tubercle. Subsequently, an oblique incision in the sartorius fascia is made to expose the hamstring tendons. The gracilis and semitendinosus tendons are carefully dissected and released distally at the tibial tubercle. The tendons are then sutured at the ends with Ethibond Excel thread to facilitate subsequent handling. The gracilis and semitendinosus are dissected and released from fascial adhesions A tendon stripper is used to release each tendon from its proximal muscular attachment while the knee is flexed in a slight varus motion.

### Graft Preparation

**Fig 1:**
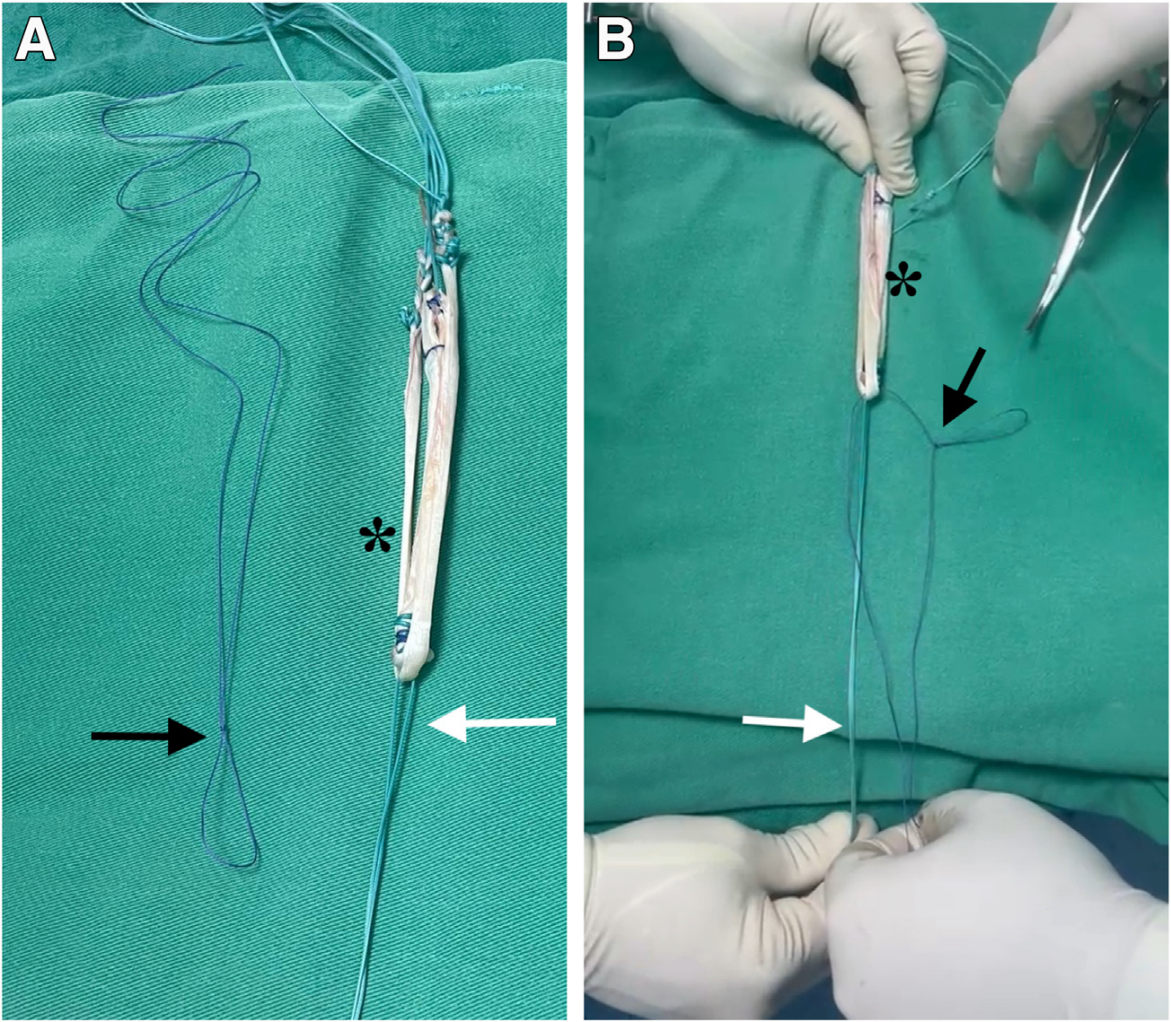
(A, B) Graft preparation. After the tendons are folded at their midpoint, an Ethibond thread (white arrows) is passed through the loop formed by the folded graft. This Ethibond thread is used to pull the graft through the tibial and femoral tunnels. A second Ethibond thread (black arrows) is passed through the flexor graft loop. This second Ethibond thread has a unique characteristic: A loop is created in it, through which the iliotibial tract is transported after the graft has passed through the femoral tunnel. The asterisks indicate the hamstring tendon grafts: gracilis and semitendinosus.

The autograft obtained is placed on the preparation table, where excess muscle tissue and damaged portions of the tendons are removed (Fig 1). The ends of the tendons are then sutured with Ethibond thread. Each tendon is folded in half to create 4 strands. After the tendons are folded at their midpoint, an Ethibond thread is passed through the loop formed by the folded graft. This Ethibond thread is used to pull the graft through the tibial and femoral tunnels. At this stage, one of the key steps in graft preparation is performed. A second Ethibond thread is passed through the flexor graft loop. This second Ethibond thread has a unique characteristic: A loop is created in this second Ethibond thread, through which the ITT graft, harvested for use in the modified Lemaire tenodesis, is passed after the hamstring graft has traversed the femoral tunnel.

A No. 0 Vicryl suture is used to transversely secure the bundles of the hamstring tendons, preventing slippage. The length and diameter of the graft are measured. After graft preparation, the arthroscopic portion of the procedure begins through the anteromedial and anterolateral portals.

### Identification of Tibial Tunnel

The center of the tibial tunnel should be at its native insertion, approximately aligned with the posterior edge of the anterior horn of the lateral meniscus, about 15 to 20 mm anterior to the posterior cruciate ligament.^8^ We use a tibial guide set to 55° and positioned with its intra-articular aiming tip, through the anteromedial portal, at the center of the ACL footprint. Extra-articularly, through the same access used for hamstring graft harvest, the guide is adjusted so that the center of the tunnel is approximately 4 cm from the tibial joint line and 2 cm medial to the tibial tuberosity. A 2-mm guide pin is then advanced through the guide until the tip is visible intra-articularly.

### Preparation of Modified Lemaire

**Fig 2:**
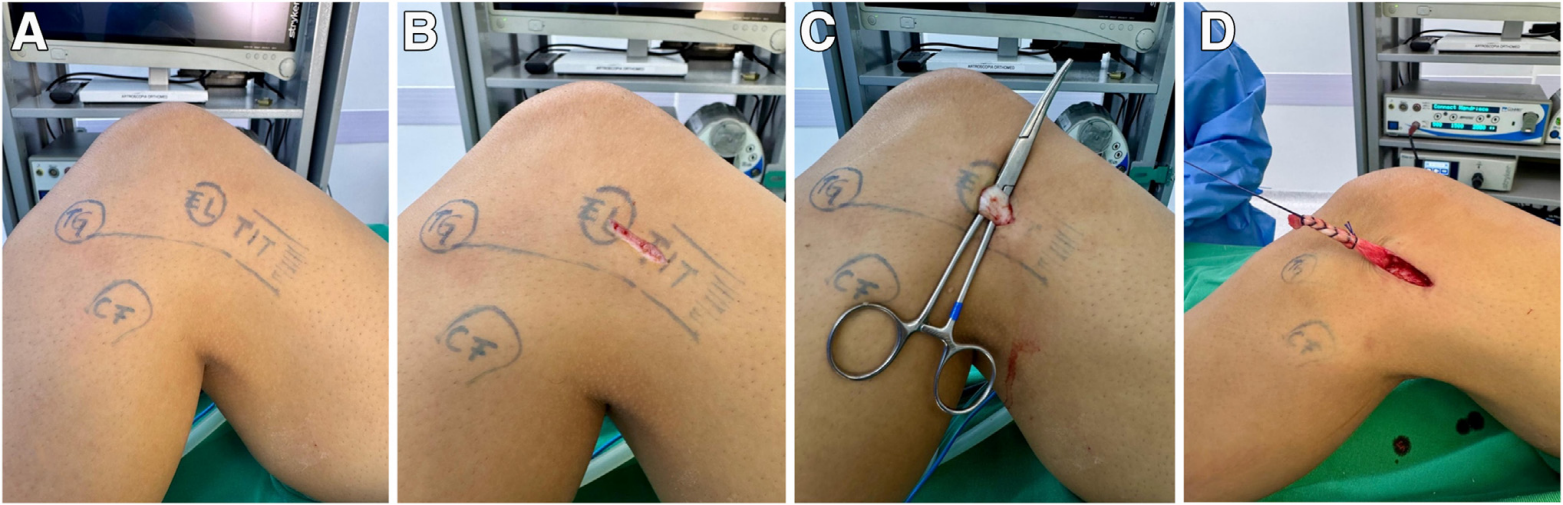
Lateral femoral access. (A) The anatomic landmarks consist of the Gerdy tubercle (TG), fibular head (CF), lateral epicondyle (EL), and iliotibial tract (TIT). (B) An incision is made on the lateral aspect of the knee, proximal to the Gerdy tubercle, extending proximally along the lateral surface of the distal femur at the level of the lateral epicondyle. (C) A strip is harvested from the middle third of the iliotibial tract, approximately 1 cm wide and 8 cm long. (D) The proximal stump of the iliotibial tract strip is sutured with absorbable No. 0 Vicryl.

An incision is made on the lateral aspect of the knee, proximal to the Gerdy tubercle, extending proximally along the lateral surface of the distal femur at the level of the lateral epicondyle (Fig 2). Variations in length and position of the incision may be necessary depending on associated procedures and the thickness of the subcutaneous fat layer. Subcutaneous tissue is dissected to adequately expose the ITT, from the Gerdy tubercle to approximately 2 cm proximal to the lateral epicondyle.

A strip is harvested from the middle third of the ITT, approximately 1 cm wide and 8 cm long. The strip is carefully released from lateral adhesions, with care taken not to damage adjacent structures, such as the joint capsule, lateral collateral ligament (LCL), or capsulo-osseous layer of the ITT, while maintaining its distal insertion on the Gerdy tubercle. The proximal stump of the ITT strip is sutured with absorbable No. 0 Vicryl.

### Identification of Femoral Tunnel

The center of the femoral tunnel is at its native insertion, in the lateral femoral condyle, posterior to the resident’s ridge.^8^ We use a 90° femoral guide, positioned with its intra-articular aiming tip through the anterolateral portal, at the center of the ACL footprint. Extra-articularly, through the lateral femoral access at the topography of the lateral femoral epicondyle, the guide is adjusted so that the center of the tunnel is near the femoral origin of the ALL, posterior and proximal to the lateral femoral epicondyle, about 4 mm and 8 mm, respectively.^9^, ^10^, ^11^ A 2-mm guide pin is then advanced through the guide until the tip is visible intra-articularly.

### Passage of Lemaire Graft Deep to LCL

**Fig 3:**
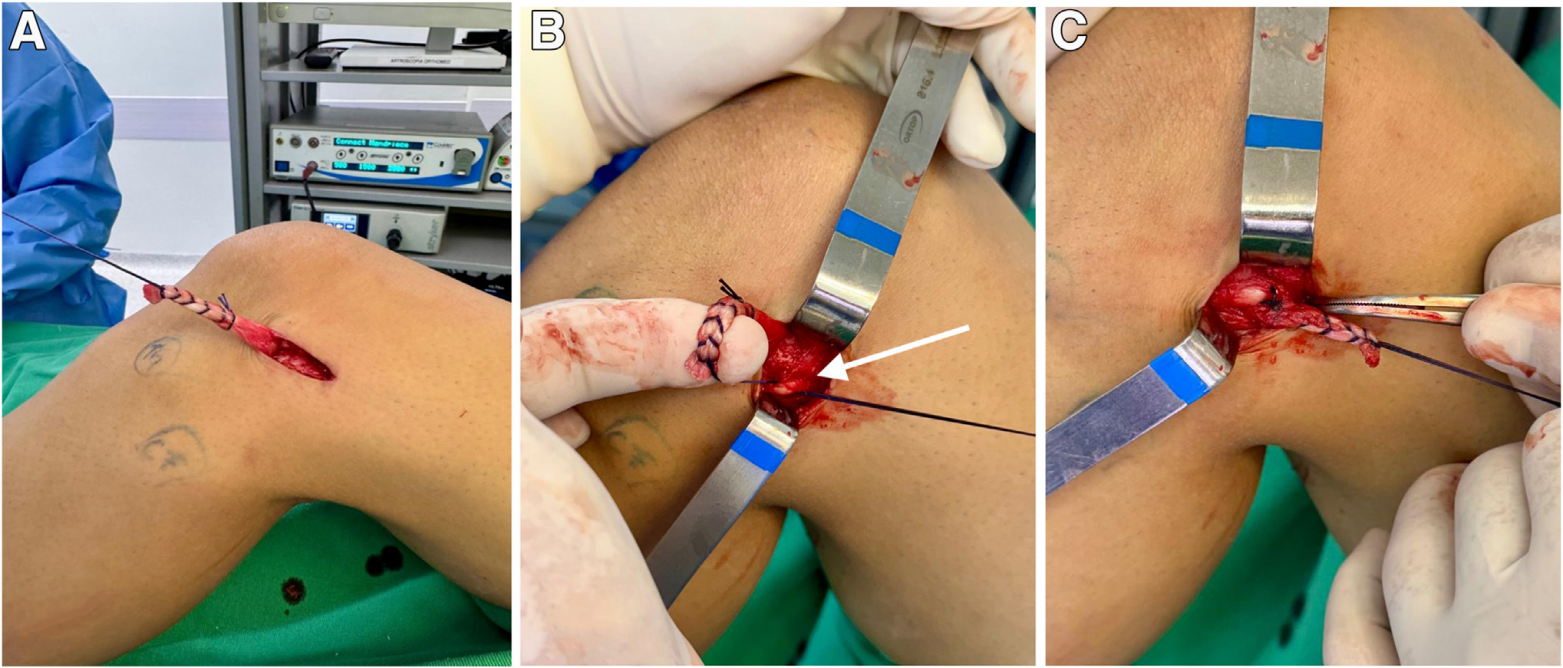
Passage of Lemaire graft deep to lateral collateral ligament. (A) The proximal stump of the iliotibial tract strip is sutured with absorbable No. 0 Vicryl (approximately 1 cm wide and 8 cm long). (B) The iliotibial tract graft is passed from anterior to posterior. The white arrow indicates the lateral collateral ligament. (C) The Lemaire graft is passed deep to the lateral collateral ligament.

After the location of the femoral tunnel is determined with the guide pin, a small longitudinal incision is made along the posterior edge of the LCL to allow the introduction of a hemostatic clamp (Fig 3). The clamp is carefully inserted deep to the LCL through the incision, with care taken not to damage the ligament fibers. The clamp can be palpated immediately anterior to the LCL, where another incision is made to expose its tip.

The clamp should remain close to the ligament at all times to avoid inadvertent damage to adjacent structures, such as the popliteus tendon, or penetration into the intra-articular space. By use of the clamp, the ITT graft (prepared in the previous step for the modified Lemaire technique) is passed from anterior to posterior.

### Drilling of Tunnels

With the knee positioned at approximately 90° of flexion, a cannulated reamer or drill of the same diameter as the hamstring tendon graft is used to create the femoral and tibial tunnels over the guide pins.

### Passage of Hamstring Grafts

**Fig 4:**
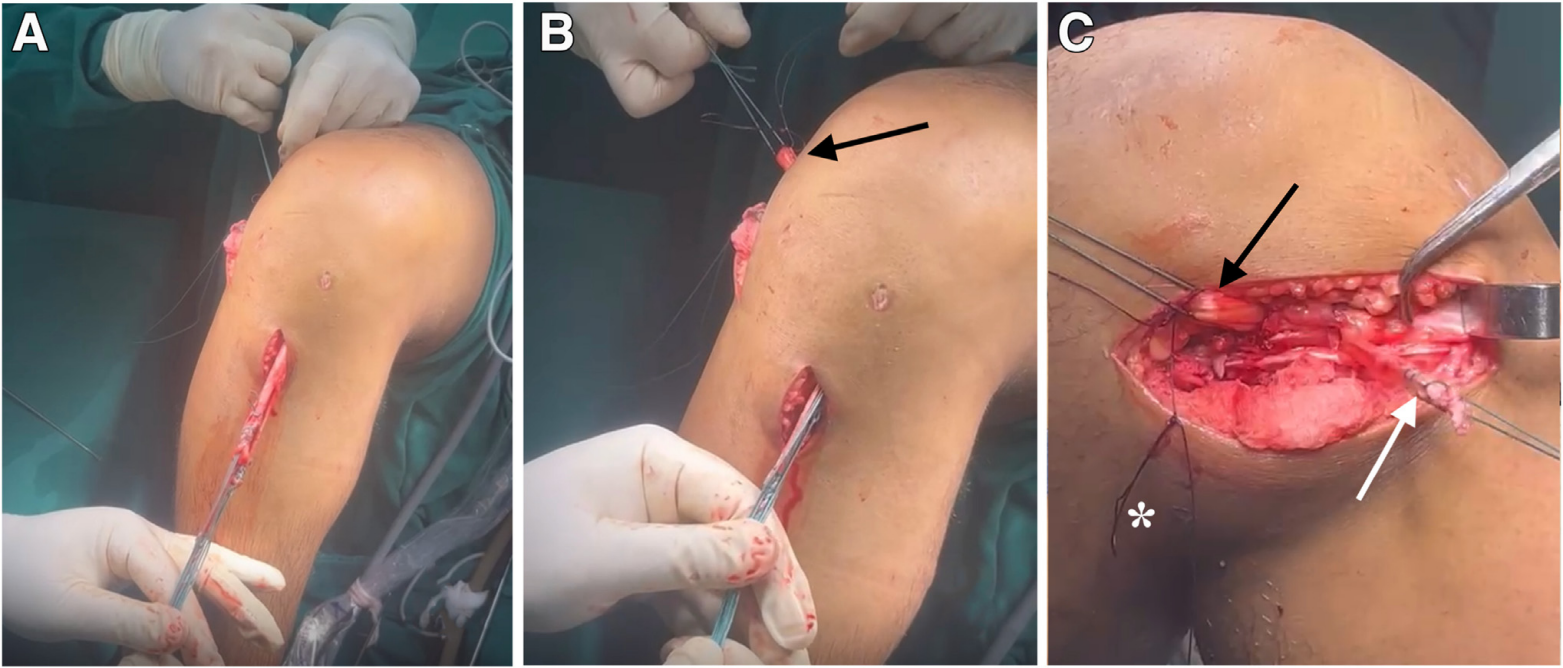
Graft passage. (A) Passage of hamstring grafts through tibial and femoral tunnels. (B) The hamstring grafts are pulled through the femoral tunnel until the graft loop (black arrow) is visible. (C) Loop of hamstring grafts through femoral tunnel (black arrow) and Lemaire graft (white arrow). As indicated by the asterisk, the second Ethibond thread is passed through the loop of the hamstring grafts (gracilis and semitendinosus).

A grasper clamp is used to pass the graft suture (the first Ethibond thread through the loop formed by the folded graft) through the tibial and femoral tunnels, making it visible in the intra-articular space, where the ACL graft will be positioned as usual (Fig 4). The graft is then pulled through the femoral tunnel until the graft loop is visible, along with the second Ethibond thread passed through the loop of the hamstring grafts.

### Passage of ITT Through the Loop of the Hamstring Grafts

**Fig 5:**
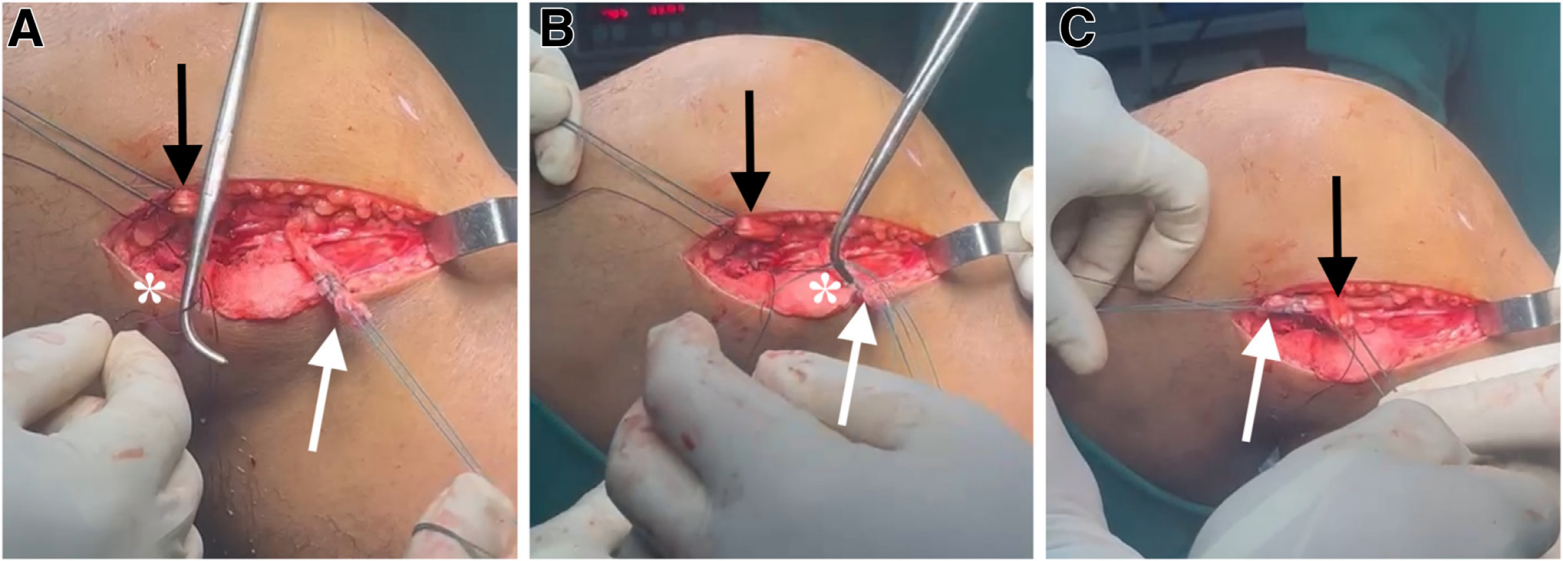
(A-C) Passage of ITT through the loop of the Hamstring Grafts. The iliotibial tract stump (white arrows), prepared in previous steps, is passed through the loop of the hamstring grafts (gracilis and semitendinosus) (black arrows) using the second Ethibond thread, forming an over-the-loop configuration, characteristic of our modified Lemaire technique. As indicated by the asterisk, the second Ethibond thread is passed through the loop of the hamstring grafts (gracilis and semitendinosus).

**Fig 6:**
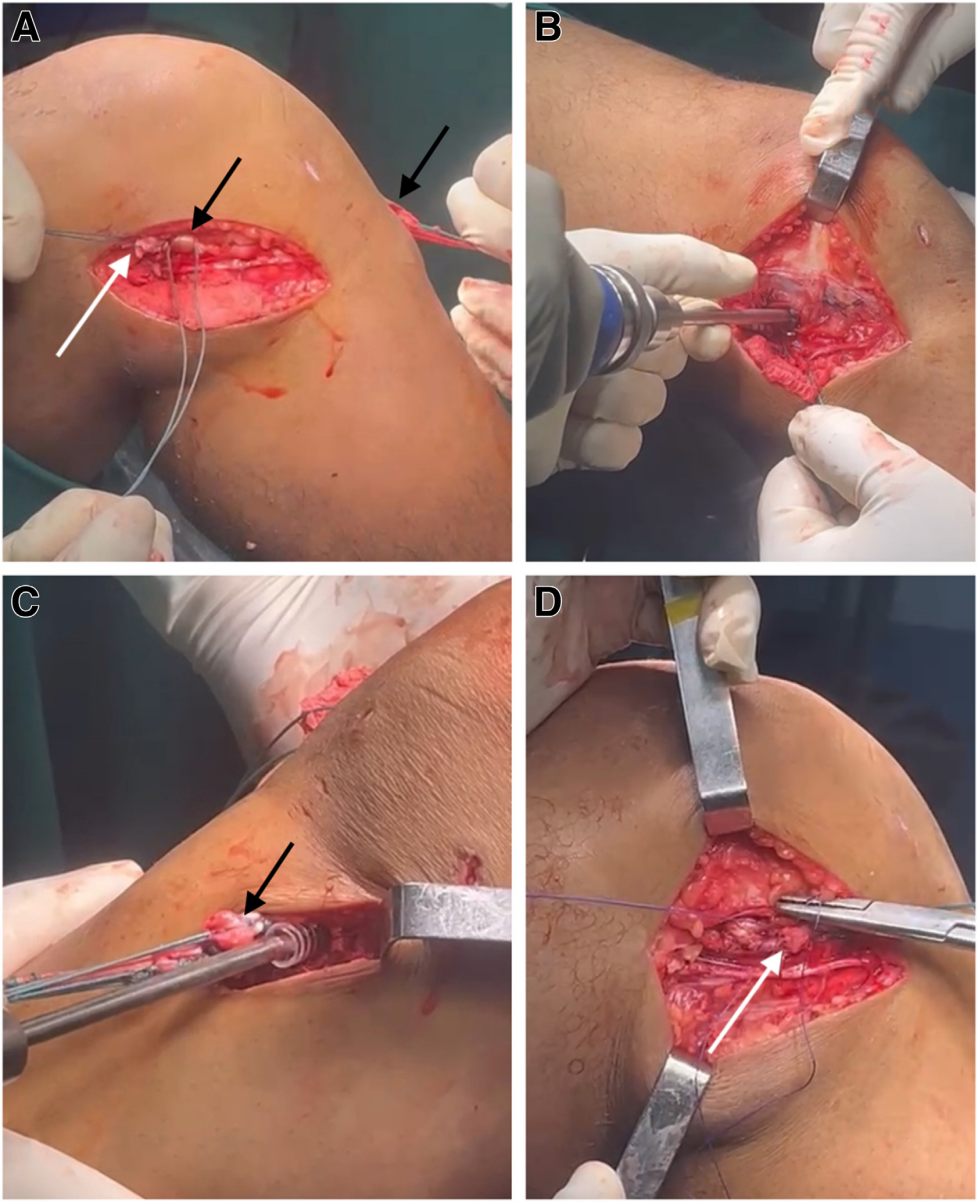
Femoral and tibial fixation. (A) Traction is applied to the graft at the tibial tunnel exit to ensure that the loop of the hamstring grafts is flush with the femoral cortex. (B) The graft is fixed in the femoral tunnel, with care taken to apply gentle traction on the iliotibial tract stump to avoid excessive tension, which could increase lateral compartment pressure postoperatively. (C) Once the graft is firmly fixed against the lateral femoral cortex with an interference screw, the knee is moved approximately 10 times to eliminate any slack in the graft. Tibial fixation of the graft is performed using an interference screw while maintaining distal traction on the graft, with the knee at 20° to 30° of flexion, in neutral varus-valgus and internal-external rotation. (D) After tibial fixation, the iliotibial tract is folded distally and sutured onto itself, completing the over-the-loop modified Lemaire procedure. The black arrows indicate the hamstring grafts, and the white arrows indicate the Lemaire graft.

The ITT stump, prepared in previous steps, is passed through the loop of the hamstring grafts using the second Ethibond thread, forming an over-the-loop configuration, characteristic of our modified Lemaire technique (Fig 5, Fig 6). Traction is applied to the graft at the tibial tunnel exit to ensure that the loop of the hamstring grafts is flush with the femoral cortex. The graft is then fixed in the femoral tunnel, with care taken to apply gentle traction on the ITT stump to avoid excessive tension, which could increase lateral compartment pressure postoperatively. Care is also taken to ensure that no part of the ACL graft protrudes from the lateral femoral condyle, which could cause friction and subsequent pain.

Once the graft is firmly fixed against the lateral femoral cortex with an interference screw, the knee is moved approximately 10 times to eliminate any slack in the graft. Tibial fixation of the graft is performed using an interference screw while maintaining distal traction on the graft, with the knee at 20° to 30° of flexion, in neutral varus-valgus and internal-external rotation. Any excess ACL graft material at the tibial end is trimmed if necessary.

### Fixation of Modified Lemaire Tenodesis and Postoperative Protocol

After the ACL graft is secured within the tibial tunnel using an interference screw, attention is turned to final fixation of the modified Lemaire tenodesis (Fig 6). The ITT, which has been passed through the loop of the hamstring graft, is now positioned along its natural course on the lateral aspect of the knee.

To ensure proper tensioning and biomechanical integrity, the ITT graft is folded distally toward its original insertion at the Gerdy tubercle. This step is crucial to prevent over-tightening, which could lead to excessive lateral compartment pressure, limiting knee range of motion postoperatively. The ITT graft is then secured onto itself using nonabsorbable sutures (No. 2 FiberWire), ensuring firm but not excessive fixation.

After reconstruction, closure is performed. No suction drain or immobilization brace is used postoperatively. Unlike some extra-articular procedures, this technique does not require prolonged external support because immediate mobilization reduces stiffness and enhances recovery.

Postoperatively, patients are allowed to bear weight as tolerated, with crutches for support during the initial phase if necessary. Formal physiotherapy begins at the first postoperative consultation (approximately 1 week after surgery), focusing on early range-of-motion exercises and quadriceps activation to prevent muscle atrophy. Progressive strength training and neuromuscular training are incorporated over the subsequent weeks to optimize return to function.

## Discussion

The presented technique combines ACL reconstruction with the over-the-loop modified Lemaire-type LET using a single femoral tunnel. This approach offers both biomechanical and practical advantages, including procedural simplification, reduced risk of tunnel convergence, and efficient use of materials, particularly in resource-limited settings.

The choice of a proximal and posterior fixation point relative to the lateral epicondyle for the mini-Lemaire reconstruction and femoral tunnel is one of the most relevant features of this technique. This positioning eliminates the risk of overlap or collapse between femoral tunnels. Furthermore, the over-the-loop passage of the ITT through the hamstring graft loop with subsequent suturing onto itself provides enhanced tension control for the extra-articular reconstruction, adding greater stability, particularly rotational, with the potential to reduce the graft rerupture rate.^7^

Hamstring grafts are advantageous owing to ease of harvest, biomechanical properties, and versatility. Retrograde tibial passage ensures proper tensioning while minimizing excess graft, reducing complications such as irritation.^12^, ^13^, ^14^, ^15^ In this technique, the retrograde passage of the grafts through the tibial tunnel facilitates adequate tensioning and allows for the removal of any excess segments without compromising graft integrity or causing friction in the femoral region. This approach minimizes the risk of complications, such as pain or irritation caused by protruding materials.

Overall, the described technique shows accessibility and reproducibility with a short learning curve. Its meticulous execution minimizes complications, while the postoperative rehabilitation follows the standard protocol for isolated ACL reconstruction, without specific adjustments for LET. These factors make this approach an attractive option, especially for high-risk patients or rotational sport athletes.

## Disclosures

All authors (C.J.S.M., R.d.C.L., C.P.H., S.M.d.G.C., P.B.J., V.B.C.d.P., D.A.d.L.) declare that they have no known competing financial interests or personal relationships that could have appeared to influence the work reported in this paper.

## Supplementary Data

Video 1 The presented technique combines anterior cruciate ligament (ACL) reconstruction and over-the-loop modified Lemaire-type lateral extra-articular tenodesis (LET) using a single femoral tunnel.

## References

[1] Hopper G.P., Pioger C., Philippe C. (2022). Risk factors for anterior cruciate ligament graft failure in professional athletes: An analysis of 342 patients with a mean follow-up of 100 months from the SANTI study group. Am J Sports Med.

[2] Moro R., Thá V.C., Ruedas V.R., Tauchmann R., Dantas G.M., Domit Filho M. (2024). Descrição de técnica de reconstrução do ligamento cruzado anterior com tenodese ântero-lateral tipo mini-Lemaire através de túnel único femoral. Rev Bras Ortop (Sao Paulo).

[3] Ariel de Lima D., Helito C.P., de Gusmão Canuto S.M. (2024). Combined reconstruction of the anterior cruciate ligament and anterolateral ligament: Triple-strand braided hamstring graft for the anterior cruciate ligament and gracilis strand for the anterolateral ligament with a single femoral tunnel. Arthrosc Tech.

[4] Daggett M., Helito C., Cullen M. (2017). The anterolateral ligament: An anatomic study on sex-based differences. Orthop J Sport Med.

[5] Hussein M., van Eck C.F., Cretnik A., Dinevski D., Fu F.H. (2012). Individualized anterior cruciate ligament surgery: A prospective study comparing anatomic single- and double-bundle reconstruction. Am J Sports Med.

[6] Ariel de Lima D., Helito C.P., Lima F.R.A.D., Leite J.A.D. (2018). Surgical indications for anterior cruciate ligament reconstruction combined with extra-articular lateral tenodesis or anterolateral ligament reconstruction. Rev Bras Ortop.

[7] Ariel de Lima D., de Lima L.L., de Souza N.G.R. (2021). Clinical outcomes of combined anterior cruciate ligament and anterolateral ligament reconstruction: A systematic review and meta-analysis. Knee Surg Relat Res.

[8] Rayan F., Nanjayan S.K., Quah C., Ramoutar D., Konan S., Haddad F.S. (2015). Review of evolution of tunnel position in anterior cruciate ligament reconstruction. World J Orthop.

[9] de Lima D.A., Helito C.P., de Lima L.L., de Castro Silva D., Cavalcante M.L.C., Leite J.A.D. (2019). Anatomy of the anterolateral ligament of the knee: A systematic review. Arthroscopy.

[10] Ariel de Lima D., Helito C.P., Lacerda de Lima L., Dias Leite J.A., Costa Cavalcante M.L. (2019). Study of the nerve endings and mechanoreceptors of the anterolateral ligament of the knee. Arthroscopy.

[11] Ariel De Lima D., Helito C.P., Daggett M. (2019). Anterolateral ligament of the knee: A step-by-step dissection. BMC Musculoskelet Disord.

[12] Helito C.P., Guimarães T.M., Sobrado M.F. (2021). Graft preparation for combined ACL and ALL reconstruction with a single femoral tunnel. Video J Sport Med.

[13] Chahla J., Menge T.J., Mitchell J.J., Dean C.S., LaPrade R.F. (2016). Anterolateral ligament reconstruction technique: An anatomic-based approach. Arthrosc Tech.

[14] Jankovic S., Vrgoc G., Vuletic F., Ivkovic A. (2021). Modified technique for combined reconstruction of anterior cruciate ligament and anterolateral ligament. Arthrosc Tech.

[15] Helito C.P., Bonadio M.B., Gobbi R.G. (2015). Combined intra- and extra-articular reconstruction of the anterior cruciate ligament: The reconstruction of the knee anterolateral ligament. Arthrosc Tech.

